# A RCT to evaluate a totally automated, culturally-adapted telephone counselor for increasing physical activity among physically inactive individuals in China

**DOI:** 10.1186/s12889-018-5386-9

**Published:** 2018-06-26

**Authors:** Lancelot W. H. Mui, Robert H. Friedman, Joseph T. F. Lau, Ji Peng, Abu S. Abdullah

**Affiliations:** 1 Division of Behavioral Health and Health Promotion, JC School of Public Health and Primary Care, The Chinese University of Hong Kong, Hong Kong, SAR China; 2 Medical Information Systems Unit, Section of General Internal Medicine, Department of Medicine, Boston University and Boston Medical Center, Boston, Massachusetts USA; 3 Shenzhen Center for Chronic Disease Control, Shenzhen, China; 4 Global Health Program, Duke Kunshan University, Kunshan, China; 5 Duke Global Health Institute, Duke University, Durham, North Carolina USA

**Keywords:** Information and communication technologies (ICT), Disease prevention, Randomized controlled trial, Diabetes

## Abstract

**Background:**

The prevalence of diabetes in China has rapidly increased in recent years. Family history and physical inactivity are known risk factors for developing diabetes. As automated telephone-based communication is recognized as a cost-effective health promoting device, the present study aims at evaluating the efficacy of an automated telephone counselor (TLC-PA-China) for promoting physical activity to the WHO-recommended level among physically inactive family members of diabetes patients.

**Methods:**

This study employed a parallel, two-group, non-blinded, randomized controlled trial design with equal allocation to the intervention group (TLC-PA-China), and a control group. Voluntary participants with at least one first-degree relative diagnosed with diabetes mellitus were recruited through eight Community Health Centers in Shenzhen, China. The intervention group was requested to use the system once per week during a six-month period. The control group received an information pamphlet about the benefits of regular physical activity.

**Results:**

Two hundred ten eligible participants were randomized to TLC-PA-China (*n* = 109) or Control (*n* = 101) groups. Using intention-to-treat analysis, the TLC-PA-China group was significantly more likely to meet the WHO physical activity recommendation than the control (GEE: OR = 6.37, *p* < 0.001). The number of physically inactive people to intervene upon for one to become active was 2.84 at 3 months and 3.31 at 6 months.

**Conclusions:**

TLC-PA-China increased physical activity levels among physically inactive adults in China who were at high risk of developing diabetes. This study lays the groundwork for application of behavioral informatics intervention in China.

**Trial registration:**

Chinese Clinical Trial Registry ChiCTR-PRC-13003361. Registered 15 May 2013 (Retrospectively registered).

## Background

The World Health Organization predicted that for the next 20 years, around 11.6% of all incident diabetes cases in the world will be in China [1]. Family history and physical inactivity are known risk factors for developing diabetics [2, 3]. Individuals who have elevated risk of developing diabetes are recommended to engage in regular physical activity [4]. In urban cities of China, such as Shenzhen, 80% of the people are physically inactive [5], 4.2% of adults have diabetes and another 11.9% have impaired glucose tolerance [6]. In Shenzhen alone, with a population of 13.2 million (as of 2012), conventional behavior change interventions that require a human counselor have only limited reach and are not easily scalable. At the same time, there are more than 20 million registered mobile phone numbers [7] in Shenzhen. Physical activity interventions that utilize telephone networks may serve to reach the enormous Chinese population with health promotion messages.

The Telephone-Linked Care (TLC) platform is a computerized, fully automatic counseling system that delivers personalized health advice and counseling to users through the telephone [8]. A recent review recognized that physical activity intervention programs are more effective when they do not involve direct supervision or instruction components [9]. The content of these automated telephone counseling programs are based on established behavioral change theories [10]. The original TLC system for physical activity was developed for use by sedentary American people [11, 12].

In this study, a TLC-based system for physical activity promotion in China [TLC-PA-China] was developed, deployed, and evaluated in a randomized clinical trial in Shenzhen, China. This study explored the efficacy of TLC-PA-China in primary prevention of diabetes among physically inactive family members of diabetes patients. The goal was to increase their physical activity level to the recommended level of at least 150 min per week of moderate-intensity aerobic activities [4].

## Methods

### Study design and setting

This study employed a parallel, two-group, non-blinded, randomized controlled trial design with equal allocation to the experimental group (TLC-PA-China), and a control group. The study was carried out through eight of the Community Health Centers managed by the Shenzhen Centre for Chronic Disease Control. The Community Health Centers provide primary care services to residents in their neighborhood.

### Participants

#### Inclusion criteria

Participants needed to have at least one first-degree relative who was diagnosed with diabetes mellitus by a physician. They must be at least 30 years of age and be physically inactive (defined as participation in less than 150 min per week of moderate or greater intensity physical activity [MOD+ PA]).

#### Exclusion criteria

People who were deemed unsuitable to participate in MOD+ PA activity by their physician were excluded from the study.

#### Recruitment of study subjects

Diabetes patients managed by the Community Health Centers were contacted by their physician and briefed about how family history and physical inactivity would increase diabetes risk of their family members. The study was introduced to the patients and invitation cards to join the study were given to those patients to take back to their immediate family members. Family members who were interested in joining contacted the Community Health Centers and were screened for physical inactivity by a research assistant using the International Physical Activity Questionnaire Short Form (IPAQ-SF) [13, 14] and by a Community Health Centre physician for their ability to perform MOD+PA. Informed consent was obtained from the participants before they joined the study.

#### Randomization

Block randomization (block size of 8) was employed during randomization. Two opaque envelopes were prepared by a trained field worker, one containing a card for the intervention group (TLC-PA-China) and the other containing a card for the control group. Participants were asked to draw an envelope after they consented to enroll into the study and were assigned to the group as indicated by the card inside the envelope.

#### Sample size

A previous TLC-PA study [12], which is also a 6 month, two-group randomized clinical trial (TLC-PA vs. control) performed in the U.S. in a sedentary general population who were patients at a multi-specialty, multi-location medical practice documented a difference between MOD+PA at 6 months follow-up favoring TLC-PA (185 ± 154 vs. 111 ± 155 min, *p* < 0.01) [11]. After controlling for gender and baseline activity level, 80% statistical power to detect this difference with a one-tailed *p*-value of 0.05 can be obtained with a sample size of 55 per group. We used a one-tailed test as we hypothesized that the intervention group would increase physical activity as compared to the control group. Since the TLC-PA-China system is a novel technology to deliver a public health intervention in China, we over-sampled by a factor of two to accommodate for attrition. This sample size corresponds to a statistical power of 97%, given that the other conditions were unchanged.

### Interventions

#### Intervention group

Participants who were randomized to the intervention group received the TLC-PA-China program and were requested to call the system once per week during a six-month period.

The TLC-PA-China system used the TLC technology to simulate face-to-face interactive counseling by a trained behavioral counselor [8]. At the beginning of each call, users entered their physical activity level for the past week. TLC-PA-China then used the digital profile (e.g. socio-demographic characteristics, behavioral constructs such as stage of change and self-efficacy for physical activity, and the person’s prior responses during the same and previous TLC conversations) of each participant in its database to provide individually-tailored utterances. At the end of each call, users participated in interactive goal-negotiation and goal-setting with the TLC system (see Table 1 for an example). TLC-PA-China conversations typically lasted between 5 to 10 min. More detailed description of the TLC system can be found in a previous paper [8]. Translation of the scripts into Mandarin Chinese was performed by the bilingual members of the research team. The content was modified to include local information that are relevant to Shenzhen, China.

**Table 1:** Example Script for a TLC-PA-China Call

1. {Call Received} 2. Introduction: “It’s time to focus on another important part of managing your health: regular exercise. Participating in regular physical activity can provide many health benefits including weight loss, lower blood pressure, improved flexibility, and increased energy. If you already exercise regularly, that’s great news. You’ll be hearing about strategies that can help you keep up the good work. If you don’t currently exercise, the information you’ll hear will focus on your motivation level. Let me explain how these calls will work. Before you make each call, you’ll need to figure out your current level of exercise. To do that, you’ll need to keep track of two things: the number of days you engaged in moderate-intensity exercise; and the average number of minutes you exercised on each of those days. At the beginning of each call, you’ll be asked to report your current level of exercise. You will need to decide how you will keep track of this information. One way to do this is to keep an exercise journal. Another way to do this is to write this information down on a calendar. When you finish giving us information about your current level of exercise, you’ll hear feedback appropriate for your level of motivation. You’ll also be asked to set an exercise goal to work on for the upcoming week, so give that some thought between calls. Each call ends with a suggestion that can help you become more active. With all of that in mind, let’s get started!” 3. Exercise frequency assessment: “Now, I’m going to ask you how often you exercised this past week. How many days did you exercise during the past week? Include any exercise that you did for 10 min or more at moderate or vigorous intensity.” 4. [Subject press “0” using telephone keypad] 5. Confirmation of answer**:** “You said you didn’t exercise at all last week. Is that correct? Please press "1″ for yes, press "2″ for no.” 6. [Subject press “1” using telephone keypad] 7. Confirmation of answer**:** “You’ve told me that you’re not currently active.” 8. Stages of change assessment**:** “Are you intending to begin exercising or to increase your exercise in the near future, say within the next 6 months? Press "1″ if you are intending to begin exercising or to increase your exercise within the next 6 months. If you don’t intend to become more active during this time, press "2″.” 9. [Subject press “2” using telephone keypad] 10. {TLC-PA-China system records that the caller is in Precontemplation stage} 11. {Call ends}	

#### Control group

Individuals randomized to the control group received an information pamphlet about the benefits of regular physical activity when they entered the study.

Outcomes and Measures.

All participants were surveyed using a structured questionnaire at baseline, 3 months, and 6 months after joining the study. The primary outcome of interest was self-reported MOD+PA level measured using the Chinese IPAQ-SF. The IPAQ-SF was shown to have acceptable reliability (ICC = 0.79) and validity (comparable to physical activity log) [14]. Other variables such as socio-demographics, physical health, and smoking status were also measured using a structured questionnaire at baseline by trained research assistants at the Community Health Centers.

#### Statistical analysis

We applied the intention-to-treat principle for data analysis. Descriptive analyses were performed for key socio-demographic indicators such as gender, age, marital status, employment type, smoking behavior and perceived economic status. The efficacy of the TLC-PA-China system to increase participants’ physical activity to the WHO-recommended level was assessed by calculating the relative risks (RR), absolute risk increase (ARI), relative risk increase (RRI), and the number needed to treat (NNT) to increase a participant’s PA level to the WHO-recommended level.

Generalized estimating equations (GEE) were used to model longitudinally the effect of the TLC-PA-China system on MOD+PA levels (linear response, exchangeable working correlation matrix), and on meeting the WHO physical activity recommendation (binary response, exchangeable working correlation matrix). Both models were controlled for the effects of baseline MOD+PA level, gender and age. All statistical analyses were performed using SPSS for Windows 18.0.

## Results

**Fig. 1:**
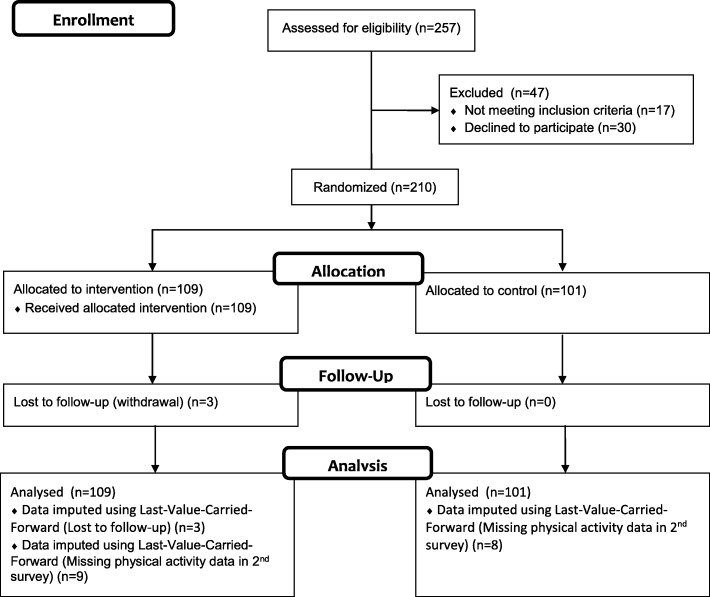
Participant Flow

Figure 1 shows the flow of participants at screening, group randomization, and the two follow-up time points. Two hundred and ten participants were randomized into the two study groups with 51.9% (109/210) in the TLC-PA-China group and 48.1% (101/210) in the control group. At baseline there was no significant difference between the study groups in their socio-demographic characteristics except that the proportion of males who were smokers was substantially higher than females (Table 2). Likewise, the study groups’ physical health status, in terms of blood pressure, body mass index, and waist-to-hip ratio were also comparable (Table 3). The males, however, showed a higher level of blood pressure and were more obese than the female participants (Table 3).

**Table 2:** Socio-demographic characteristics by treatment group or gender

	% or Mean (SD)						
	All (*n* = 210)	Intervention (*n* = 109)	Control (*n* = 101)	p*	Male (*n* = 76)	Female (*n* = 134)	p*
Gender				NS			
Male	36.2	36.7	35.6				
Female	63.8	63.3	64.4				
Age (year)	41.4 (7.20)	41.9 (7.36)	40.9 (7.03)	NS	40.0 (7.89)	42.2 (6.69)	0.036
Education level				NS			NS
Primary or below	1.4	0.9	2.0		1.3	1.5	
Junior high	7.1	7.3	6.9		6.6	7.5	
Senior high	39.5	41.3	37.6		38.2	40.3	
Junior college	28.1	24.8	31.7		21.1	32.1	
University or above	23.8	25.7	21.8		32.9	18.7	
Marital status				NS			NS
Single	5.7	6.4	5.0		7.9	4.5	
Married	85.2	85.3	85.1		80.3	88.1	
Separated	0.5	0.9	0.0		1.3	0.0	
Divorced	7.6	7.3	7.9		10.5	6.0	
Widowed	1.0	0.0	2.0		0.0	1.5	
Employment type				NS			0.037
Full-time	69.5	69.7	69.3		78.9	64.2	
Part-time or temporary	9.0	10.1	7.9		10.5	8.2	
Unemployed	10.5	10.1	10.9		7.9	11.9	
Retired	7.6	7.3	7.9		1.3	11.2	
Others	3.3	2.8	4.0		1.3	4.5	
Smoking status				NS			< 0.001
Never smoker	76.2	77.1	75.2		36.8	98.5	
Current smoker	17.6	16.5	18.8		47.4	0.7	
Ex-smoker	6.2	6.4	5.9		15.8	0.7	
Doctor-rated economic status				NS			NS
Rich	2.4	2.8	2.0		0.0	3.7	
Comparatively rich	22.4	20.2	24.8		21.1	23.1	
Average	60.5	62.4	58.4		63.2	59.0	
Below average	12.9	14.7	10.9		14.5	11.9	
Poor	0.5	0.0	1.0		0.0	0.7	
Don’t know	1.4	0.0	3.0		1.3	1.5	
Self-rated economic status				NS			NS
Rich	0.5	0.9	0.0		0.0	0.7	
Comparatively rich	12.4	11.9	12.9		6.6	15.7	
Average	63.8	64.2	63.4		64.5	63.4	
Below average	21.4	21.1	21.8		26.3	18.7	
Poor	1.9	1.8	2.0		2.6	1.5	
Pay out-of-pocket for medical				NS			0.046
No	1.4	1.8	1.0		3.9	0.0	
Yes	98.6	98.2	99.0		96.1	100.0	

Study was conducted at Shenzhen, China between July and August 2009

NS: *p* < 0.05

* Student’s t-test for *age*; Fisher’s Exact Test for *Pay out-of-pocket for medical*, Pearson Χ^2^ for all others

**Table 3:** Baseline physical health by treatment group or gender

	% or Mean (SD)				% or Mean (SD)		
	All (*n* = 210)	Intervention (*n* = 109)	Control (*n* = 101)	p*	Male (*n* = 76)	Female (*n* = 134)	p*
Systolic blood pressure (mmHg)	113.8 (13.03)	114.8 (13.84)	112.8 (12.09)	NS	118.2 (10.80)	111.4 (13.57)	< 0.001
Diastolic blood pressure (mmHg)	75.4 (9.17)	75.8 (9.87)	74.9 (8.38)	NS	78.2 (7.38)	73.8 (9.71)	< 0.001
Heart rate (bpm)	72.8 (7.30)	72.3 (7.43)	73.4 (7.14)	NS	73.6 (7.00)	72.3 (7.45)	NS
Height (cm)	162.5 (7.33)	162.9 (6.99)	162.1 (7.68)	NS	169.4 (5.78)	158.6 (4.79)	< 0.001
Weight (kg)	61.7 (10.83)	62.3 (11.53)	61.1 (10.03)	NS	70.2 (10.69)	56.9 (7.40)	< 0.001
Waist circumference (cm)	81.6 (9.68)	81.8 (10.75)	81.5 (8.43)	NS	87.7 (9.76)	78.2 (7.78)	< 0.001
Hip circumference (cm)	95.5 (6.66)	95.7 (6.48)	95.3 (6.87)	NS	97.9 (7.49)	94.2 (5.76)	< 0.001
Body mass index (kg/m^2^)	23.3 (2.94)	23.4 (3.19)	23.1 (2.66)	NS	24.4 (3.10)	22.6 (2.65)	< 0.001
Waist-to-hip ratio	0.85 (0.643)	0.85 (0.07)	0.85 (0.06)	NS	0.89 (0.052)	0.83 (0.058)	< 0.001
Obesity status				NS			< 0.001
Normal weight	50.5	51.4	49.5		34.2	59.7	
Overweight ([23–25) kg/m^2^)	23.2	22.0	24.8		26.3	21.6	
Obese (> = 25 kg/m^2^)	26.2	26.6	25.7		39.5	18.7	

Study was conducted at Shenzhen, China between July and August 2009

NS: *p* >0.05

* Pearson Χ^2^ for *obesity status*, Student’s t-test for all others

Although all study subjects had less than 150 min MOD+PA measured at baseline by the IPAQ-SF, 19 of those who were randomized to the intervention group reported over 150 min per week of MOD+ PA to the TLC system during their first TLC call. Nonetheless, we retained all 19 subjects in the study. Differences between TLC-measured and IPAQ-measured physical activity are expected because 1) baseline administration of the IPAQ-SF preceded the first TLC call with the subjects by days, and 2) TLC assessed physical activity differently than the IPAQ-SF. We dealt with missing data using the last-value-carried-forward approach which provided a conservative estimate of the effect.

**Fig. 2:**
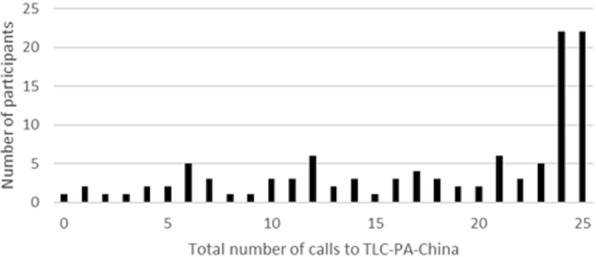
TLC-PA-China Utilization

**Fig. 3:**
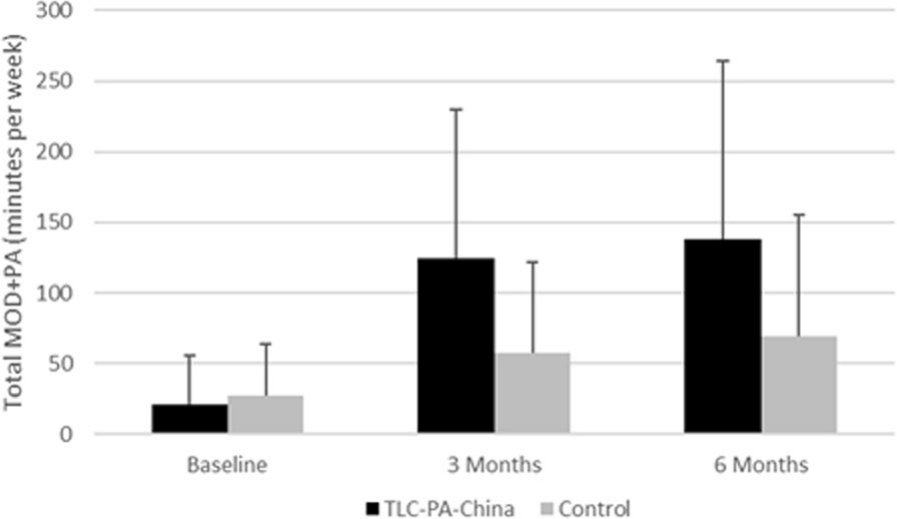
Time Spent on MOD+PA

Forty percent (44/109) of the participants in the TLC-PA-China group received the full dose of intervention by calling the system at least 24 times over the six-month period (Figure 2). Median number of calls to the system was 21 times. Weekly time spent on MOD+PA for both groups is shown on Figure 3. Participants who were in the TLC-PA-China group were more likely to become physically active than the control group at both 3 months (RR = 6.09, 95% CI = 2.99, 12.79; ARI = 35.27%; RRI = 508.91%; Table 4) and 6 months (RR = 3.18, 95% CI = 1.91, 5.42; ARI = 30.18%; RRI = 217.69%; Table 4). The effect of the TLC-PA-China system appeared to have plateaued by 3 months and the effect remained stable at 6 months. In contrast, there was a weaker, but gradual, increase in the proportion of the control group participants who became physically active over the 6 months study period. The number of participants needed to treat to increase physical activity in one participant to the WHO-recommended level was 2.84 at 3 months and 3.31 at 6 months (Table 4).

**Table 4:** Effectiveness of TLC-PA-China to promote physical activity

		> = 150 min/wk. at least moderate intensity physical activity (%)		
Group	N	Baseline	3 months	6 months
Control	101	0.0	6.9	13.9
Intervention	109	0.0	42.2	44.0
			RR (95% CI) = 6.09 (2.99, 12.79)	RR (95% CI) = 3.18 (1.91, 5.42)
			ARI = 35.27%	ARI = 30.18%
			RRI = 508.91%	RRI = 217.69%
			NNT = 2.84	NNT = 3.31

Study was conducted at Shenzhen, China between July and August 2009

*RR* Relative Risk, *ARI* Absolute Risk Increase, *RRI* Relative Risk Increase, *NNT* Number Needed to Treat

The generalized estimating equation analysis showed that at 6 months, the intervention group subjects were 7.34 times more likely to meet WHO-recommended physical activity level than the control group (*p* < 0.001; Table 5), while controlling for baseline MOD+PA, gender, and age. An interaction model that adds the intervention*time interaction was also tested but the interaction term was not significant (*p* > 0.05; Table 5). For participation in MOD+PA, GEE analysis with intervention*time interaction showed that by 6 months, on average intervention group subjects participated in 12.73 more minutes/wk. of MOD+PA than control group subjects. Among control group subjects, on average there was a 5.35 min/wk. monthly increase of MOD+PA over 6 months. On the other hand, intervention group subjects increased their MOD+PA level by 19.56 min/wk. each month over the same period (Table 6).

**Table 5:** Longitudinal modeling of meeting physical activity recommendation using generalized estimating equation

	Non-interaction model			Interaction model		
	OR	95% CI	*p*-value	OR	95% CI	*p*-value
TLC-PA-China	7.34	(4.164, 12.927)	< 0.001	8.39	(2.792, 25.200)	< 0.001
Time (months)	1.53	(1.431, 1.641)	< 0.001	1.57	(1.293, 1.900)	< 0.001
TLC-PA-China*Time	–	–	–	0.97	(0.793, 1.193)	NS
Baseline MOD+PA	1.01	(1.004, 1.018)	0.003	1.01	(1.004, 1.018)	0.003
Gender	1.23	(0.702, 2.145)	NS	1.23	(0.701, 2.141)	NS
Age	1.02	(0.985, 1.065)	NS	1.02	(0.985, 1.065)	NS

Study was conducted at Shenzhen, China between July and August 2009

NS: Not significant at α = 0.05

^Note^ Results are estimated for a binary outcome with logit link function and exchangeable working correlations

The asterisks indicate which variables are involved in the interaction

**Table 6:** Longitudinal modeling of MOD+PA level using generalized estimating equation

	Non-interaction model			Interaction model		
	β	95% CI	p-value	β	95% CI	*p*-value
TLC-PA-China	55.28	(41.482, 69.087)	< 0.001	12.73	(5.105, 20.347)	0.001
Time (months)	12.74	(10.207, 15.276)	< 0.001	5.35	(2.792, 7.899)	< 0.001
TLC-PA-China*Time	–	–	–	14.21	(9.596, 18.826)	< 0.001
Baseline MOD+PA	0.76	(0.568, 0.949)	< 0.001	0.76	(0.567, 0.974)	< 0.001
Gender	11.90	(−3.271, 27.070)	NS	11.79	(−3.370, 26.957)	NS
Age	0.484	(−0.571, 1.540)	NS	0.489	(−0.566, 1.545)	NS

Study was conducted at Shenzhen, China between July and August 2009

NS: Not significant at α = 0.05

^Note^ Results are estimated for a linear outcome and exchangeable working correlations

The asterisks indicate which variables are involved in the interaction

## Discussion

Our findings showed that the TLC-PA-China system was efficacious in increasing physical activity in inactive family members of diabetes patients to levels recommended by public health authorities in China and other countries [15, 16]. The effect was substantial even though we used conservative analytic methods. Although the MOD+PA level in control group increased during the study period, possibly because of other health promotion activities in the community, the increase was substantially higher for those who used the TLC-PA-China system.

Using printed pamphlets (our control condition) to disseminate health information is a common practice because of its potential reach and ease of distribution. However, with the advancement in information and telecommunication technologies and the ubiquitous availability of the telephone, delivery of health information over the telephone has become practical. The TLC-PA-China system takes it to another level by providing not just information, but a personalized, interactive health behavior counseling intervention to the users.

With the increasing privatization of the health care industry in China and decreasing health care funding from the Government [17], health care providers have increased the charges for their service in order to stay solvent, and resulted in substantially higher out-of-pocket payments by the patients [18]. Large out-of-pocket costs have become the primary reason for people to refuse medical care services recommended by their physician [17]. There is a very pressing need in China for health promotion and disease prevention programs that are easy to deploy and cost-effective. For every four people who were assigned to use the TLC-PA-China system in our study, one previously physically inactive person became active. If this level of physical activity can be sustained by the TLC users after the study, TLC use would have helped reduce the risk of diabetes in a substantial proportion of the participants [3]. Although the TLC-PA-China system was only tested in an urban setting, the increasing urbanization in China and other countries makes this a very promising approach. In addition, given the importance of physical activity in reducing the growing burden of diabetes and obesity in rural settings, this TLC-PA-China prototype should also be adapted and tested in rural settings. While they do not usually have access to health promotion experts nearby, at least 96% of people living in rural villages in China have access to mobile telephones [19]. In addition, the use of the TLC-PA-China system does not require the user to be able to read Chinese, which is advantageous in rural China where as many as 65% of the population is unable to read [20]. Since Mandarin is the spoken language throughout the country in both rural and urban areas, TLC-PA-China’s use of spoken language eliminates, or substantially reduces, the problem of implementing public health programs using printed materials. Implementation of TLC-PA-China in both urban and rural areas in China with very large numbers of at-risk relatives of diabetes patients would dramatically improve the cost-effectiveness of the intervention. Most of the costs of a TLC system are capital costs for the system’s computer and telecommunication hardware and software, but the operating cost of the system is very low and requires minimal manpower support [8].

The use of pre-recorded messages improves the fidelity of the intervention since it removes the known variability in the performance and quality of human counselors [21, 22]. Automatic information hotline systems are often criticized for their inflexibility and lack of options for user feedback. The TLC-PA-China system is tailored to the user’s needs and is always available to them. The rapid adoption of mobile phones in China, from only 6.72 lines per 100 inhabitants in 2001 [23] to 73.6 lines per 100 inhabitants in 2012 [24] allows most people in China to access the TLC-PA-China system. The ability to deliver the program through this accessible and inexpensive platform makes it practical for use in China’s public health system to address the rapid increase in sedentary behavior in the country. There is a dearth of effective interventions to fight the rapid increase in lifestyle-related chronic disease in China and other developing countries [25, 26]. The TLC-PA-China approach could also be used for changing other potentially modifiable behavioral risk factors for disease (e.g. cigarette smoking and unhealthy diets).

The current study was performed in a single city in China with a relatively small group of individuals who were at higher risk of developing diabetes than the general population. After this efficacy study, the next logical step is to evaluate TLC-PA-China in an effectiveness study to determine how it functions in actual practice, and eventually in real world implementation in different settings and populations across China and beyond.

The main drawback of any automated system, such as the TLC platform, is the inability of the system’s designers to anticipate all the issues and situations that users face. This has not been practically important since the health behavior change models used in TLC programs cover the principal factors that affect a person’s health behavior and the principal situations that humans find themselves in with respect to their health behavior [27]. Another potential limitation of the TLC-PA-China system is the reliance on user self-report of physical activity. In future implementation of the system in China, an objective measure of physical activity such as a pedometer or accelerometer can be used [12]. Participants in this study expressed interest in participating, so the results may not generalize to individuals who are not motivated to change their physical activity behavior. Finally, the long-term effectiveness of the TLC-PA-China system is still unclear because the program only ran for 6 months. However, in a U.S. study of TLC-PA, intervention effects of a 6-month intervention were sustained at 12 and 18 months follow up [11, 28].

## Conclusions

This study introduced the behavioral informatics approach to health promotion into China. TLC-PA-China is an effective and low-cost intervention to increase the physical activity level of people at higher risk of developing diabetes in China. The monetary and time costs to participants are minimal because the automated system uses the existing telephone network to deliver the intervention with around-the-clock access. Further studies are needed to determine the long-term effectiveness of this approach, as well as to explore the effectiveness of applying this technology in routine practice in both urban and rural China. Application of the TLC platform for other behavioral health interventions in China also warrants further exploration [29, 30]. The popularity of smartphones opens up future opportunities for the TLC platform to interact with smartphone users through interactive apps.

## Abbreviations

ARI: Absolute risk increase
GEE: Generalized estimating equations
IPAQ-SF: International physical activity questionnaire short form
MOD+ PA: Moderate or greater intensity physical activity
NNT: Number needed to treat
RR: Relative risks
RRI: Relative risk increase
TLC: Telephone-linked care
TLC-PA-China: TLC-based system for physical activity promotion in China
WHO: World Health Organization
